# SHORT-TERM WEALTH CHANGES AND SUBSEQUENT COGNITIVE HEALTH AMONG OLDER ADULTS IN CHINA, ENGLAND, MEXICO, AND THE US

**DOI:** 10.1093/geroni/igac059.410

**Published:** 2022-12-20

**Authors:** Tsai-Chin Cho, Xuexin Yu, Alden Gross, Yuan Zhang, Lindsay Kobayashi

**Affiliations:** University of Michigan School of Public Health, Ann Arbor, Michigan, United States; University of Michigan, Ann Arbor, Michigan, United States; Johns Hopkins Bloomberg School of Public Health, Baltimore, Maryland, United States; University of North Carolina at Chapel Hill, Chapel Hill, North Carolina, United States; University of Michigan, Ann Arbor, Michigan, United States

## Abstract

Household wealth is positively associated with later-life cognitive health, but little is known about the effects of changes in wealth over time and whether they differ across populations. In this study, we evaluated the within- and between-country relationships between short-term changes in household wealth and subsequent cognitive function among adults aged ≥65 years in China, England, Mexico, and the US. We used sampling-weighted, multivariable-adjusted linear models to estimate the relationships between household wealth change over 3- to 4-year periods and subsequent harmonized general cognitive performance factor scores using HCAP measures. We found that short-term decreases in household wealth were associated with poor subsequent cognitive health in the US and China, but not in England or Mexico. The observed associations were weaker in Mexico than in the US. In summary, macro-level social and economic structures may modify the association between wealth changes and cognitive health, although further investigation is needed.

